# Effect of β-glucan on the immune response of early stage of *Anabas testudineus* (Bloch) challenged with fungus *Saprolegnia parasitica*

**DOI:** 10.1186/2193-1801-2-197

**Published:** 2013-04-30

**Authors:** Basanta Kumar Das, Pranati Pattnaik, Chandan Debnath, Dipak Kumar Swain, Jyotirmayee Pradhan

**Affiliations:** Fish Health Management Division, Central Institute of Freshwater Aquaculture (CIFA), P. O. Kausalyagnaga, Bhubaneswar, Odisha 751 002 India

**Keywords:** *Anabas testudineus*, *Saprolegnia parasitica*, β-glucan, Immunity, Bactericidal

## Abstract

The present study was carried out to study the effect of different dosages of β-glucan suspension on immune response and disease resistance in *Anabas testudineus* spawns against a fungal pathogen *Saprolegnia parasitica*. Eight day old spawns were exposed for 3 h in four different dosages of β-glucan suspension in phosphate buffered saline at the rate of 0, 5, 10, 15 mg l^-1^. The cell suspension of spawn was assayed for total protein, acid phosphate, lysozyme, bactericidal and NBT activity. The spawns were then challenged with 3x10^5^ CFU ml^-1^ of *S. parasitica*. The survivability percentage and immunological parameters were assayed upto day 7. Exposure of fish for 3h to all the concentrations of β-glucan recorded a significant enhancement in the immunological parameters such as lysozyme activity, bactericidal activity and NBT activity by the end of day 7. The challenge study indicated least mortality in the groups exposed to 10 mgl^-1^ and 15 mgl^-1^ but 15 mgl^-1^ gives little higher survivability. Thus 3 h exposure of β-glucan suspension could reduce the mortality and increase the immunity of *A. testudineus* spawns against *S. parasitica*.

## Introduction

*Saprolegnia* is ubiquitous in freshwater ecosystems and is considered as the main genus of water molds responsible for significant fungal infections of freshwater fish and eggs (Noga 1996), especially from the egg stage through smoltification (Bruno & Wood 1999; Pickering 1994). *Saprolegnia* is an opportunist facultative parasite (Neish 1977), which is saprotrophic and necrotrophic (Bruno & Wood 1999). On fish, *Saprolegnia* invades epidermal tissues visible as white or grey patches of filamentous mycelium (Bruno & Wood 1999; Beakes et al.^1994^), generally beginning on the head or fins (Neish 1977; Willoughby 1994) and can spread over the entire surface of the body. *Saprolegnia* is characterized by an external, cotton-like appearance that radiates out in a circular, crescent-shaped or whorled pattern. The fungal spores may be transmitted by hatchery fish, wild fish, eggs, water supplies, and equipment (Bruno & Wood 1999).

A number of workers have reported *Saprolegnia* infection in fishes; salmonids (Beakes et al. 1994; Hatai & Hoshiai , Hatai & Hoshiai Hatai & Hoshiai 1994), teleosts (Bruno & Wood 1999), Channel catfish (Howe et al. 1999), pike (Willoughby 1985), bass (Noga 1996), elver and suckers (Roberts 1989), roach, orfe, carp, tench, lamprey, sturgeon, barramundi, tilapia, and mullet (Bruno & Wood 1999). It has also been associated with tropical fish, including the kissing gourami, guppy, swordfish and platyfish (Willoughby 1994; Roberts 1989). The air breathing teleost, *Anabas testudineus*, from an Indian river was also found to carry *S. parasitica* infections (Mohanta & Patra 1992).

*Saprolegnia* generally invades fish that have been stressed or otherwise have weakened immune systems (Bruno & Wood 1999). A number of chemicals such as malachite green (Willoughby & Roberts 1992; Bruno & Wood , Bruno & Wood Bruno & Wood 1999), 37% formaldehyde (Van Waters 1988), hydrogen peroxide (Fitzpatrick et al. 1995; Marking et al. 1994), sodium chloride at high concentrations (Pickering 1994;Marking et al. 1994), etc. are available for treatment of fungal infection but a few are approved for use in aquaculture. The reduction of stress and boosting up immune response by application of immunostimulants appears to be the single greatest factor to help fish resist saprolegniasis.

β-glucan are a group of glucose polymers which are the main structural components of cell-wall in fungi, plants and some bacteria (Brown & Gordon 2003). They can be derived from the cell walls of yeast, bacteria, fungi, and cereals such as oats, barley, and rye. They have a backbone of β (1–3)-linked β -D-glucopyranosyl units with side chains of different lengths. A variety of cell surface receptors bind β -glucan, including lectins, scavenger receptors, and integrins on monocyte/macrophages, neutrophils, and natural killer (NK) cells and various lymphocyte subpopulations (Brown & Gordon 2003). Engagement of these receptors by β -glucan may induce activation of leukocytes, phagocytic activity, production of inflammatory cytokines and chemokines, microbial killing, and initiate the development of adaptive immunity, all of which contribute to the anti-infective and antitumorigenic properties of β -glucan (Brown et al. 2003; Misra *et al.*^2006^). β -Glucan has also been shown to enhance resistance of fish and crustacea against bacterial and viral infections (Chang et al. 2003; Misra et al. 2004; Misra et al. 2006).

The present experiment was designed to study the effect of β –glucan immersion in three different doses on immune response and survival of eight day old spawn of *Anabas testudineus* challenged with *Saprolegnia parasitica*.

## Materials and methods

### Fish

*Anabas testudineus* spawns of 8 days old were obtained from the farm of Central Institute of Freshwater Aquaculture (CIFA), Bhubaneswar, Orissa, India. The spawns were acclimatized in a circular plastic container of 20 l capacity in the laboratory of Fish Health Management Division, CIFA for two days. The spawns were fed with a formulated diet obtained from CIFA at 5% of the total body weight for two times a day.

### β–glucan

β–glucan obtained from Barley (Sigma) in powder form was added to phosphate buffer saline (pH 7.4) and subsequently sonicated (Artek Sonic Dismembrator Model 150) using a microtip at a relative output of 0.6 and duty cycle of 40%. The concentration was adjusted as 5 mgl^-1^, 10 mgl^-1^ and 15 mgl^-1^ respectively by using sterile milliQ water (Millipore Corporation, India). Immersion was selected as the route of administration of β–glucan.

### Experimental design

The spawns after acclimatization were divided into four major groups for exposure to different concentrations of β–glucan, such as Group A (0 mg l^-1^, control), Group B (5 mg l^-1^), Group C (10 mg l^-1^) and Group D (15 mg l^-1^). Fifty spawns were kept in each of the duplicate beaker (1L) for each treatment group. After bath exposure with β –glucan suspension for 3 h, four spawns were collected from each tank after a time interval of 3 h for evaluating changes in lysozyme activity, acid phosphate (AcP) activity, total protein content, bactericidal activity and superoxide anion assay. As it was difficult to draw blood from spawn with indistinctly developed internal organs, the entire body of four spawns taken at a time was aseptically placed in a macerator containing Hank’s Balanced Salt Solution (HBSS) and anticoagulant, heparin. The macerated sample was layered on to preform continuous gradients of 51% percoll (Pharmacia, Uppsala, Sweden) in 8.5 g l^-1^ NaCl and centrifuged at 800 g for 20 min at 4°C. The white cell band formed at the interface of the cell suspension and percoll layer was harvested with a Pasteur pipette, diluted ten folds with HBSS and recentrifuged at 800 g for 10 min at 4°C to remove residual percoll. The resulting pellet was again washed twice and centrifuged at 800 g for 10 min at 4°C. The resultant white band cell suspension was used for assaying lysozyme, acid phosphatase, bactericidal, superoxide anion activities and total protein content. After the third wash, the concentration and viability of leucocytes cell suspensions were determined in 0.2% trypan blue. The total protein content of the cell lysate was analysed using protein kit (Bangalore Genei Protein Kit, Bradford macro method) and bovine serum albumin as standard as per the manufacturers instructions.

### Challenge

After exposing in β–glucan suspension for 3 h, the spawns were removed from the glucan treated/untreated group and placed in normal freshwater. The spawns were divided into two sub-groups (32 nos. each) and each treatment group was challenged by immersing in viable fungal suspension of *Saprolegnia parasitica* at a concentration of 3×10^5^ CFU ml^-1^ for seven days. Four spawns from the first sub-group of each treatment challenged with *S. parasitica* were taken on day 3 and day 7 post challenges for evaluating changes in lysozyme, acid phosphate and total protein content. Each test was repeated four times and mean of the sample was taken.

### Lysozyme activity of the cell suspension

 Lysozyme activity of cell suspension in each subgroup was measured by turbidimetric method described by Parry *et al.*(1965) using 0.2 mg ml^-1^ lyophilized *Micrococcus luteus* ATCC 49732 (DIFCO, BBL- Qualis lab) as the substrate in 0.05 M phosphate buffer (pH 6.2). Various amount of cell suspension (25-100 μL) were added to 2 ml of the bacterial cell and the absorbance was measured at 0.5 and 4.5 min intervals at 530 nm (25°C). One unit of lysozyme activity is defined as the amount of sample causing a decrease in absorbance of 0.001 min^-1^.

### Bactericidal activity of the cell suspension

Bactericidal activity of the cell suspension was estimated by following the procedure of Kajita et al. (1990) with little modification. An equal volume (100 μl) of the spawn cell suspension and bacterial cell was mixed and incubated for 1h at 25°C. A blank control was prepared by replacing the cell suspension with sterile PBS. The mixture was then diluted with sterile PBS at a ratio of 1: 10. The diluted mixture (100 μl) was pour plated in nutrient agar and plates were incubated for 24 h at 37°C. The number of viable bacteria was determined by counting the colonies grown in nutrient agar plates.

Bactericidal activity in % = (No. of colonies in control- No. of colonies in sample × 100) / No. of colonies in control

## NBT activity of the cell suspension

The NBT activity was measured according to Chung & Secombes (1988) with some modifications. At first, 96-well flat bottom microtitre plate was taken and coated with 100 μl poly-L-lysine solution (0.2% Sigma). To this, 100 μl cell suspension sample was added in 5 of the 96 wells of the microtitre plate and then incubated at 25°C for 2 h and then washed with HBSS. Then 100 μl of NBT solution (0.02 gm NBT + 0.050 μl methanol + 0.94 μl PBS + 0.010 μl bacterial suspension) was added, which contained 10^6^ numbers of *A. hydrophila* cells/ml. Subsequently it was incubated at 25°C for 1h. Then the medium was removed and the reaction was terminated. Again washing was done (3 times) by addition of methanol, which was followed by air drying. Subsequently, the formazan in each well was dissolved with 120 μl of 2 M KOH and 140 μl of DMSO. The absorbance was measured at 655 nm in the ELISA reader (Biorad), with 405 nm as reference.

## Acid Phosphatase activity

The acid phosphatase activity was measured using Acid phosphatase kit (Batch No. 14407/14507 manufactured by Accurex Biomedical Pvt. Ltd.) following the method of Hillman (1971). The working solution was prepared by dissolving the contents of the substrate bottle with diluent. One ml of the AcP working solution was added to 100 μl of sample. Absorbance was measured after 5 min at 405 nm and subsequently three more readings were recorded after 1 min interval. The concentration of AcP was calculated as u/l by using the formulae i.e*.,* Conc. in 1u/l = 743 × Δ Abs./ min

### Statistical analysis

The mean values of these parameters were recorded over five days and ANOVA followed by Duncan’s multiple range tests were performed on the data (SAS version 9.1) to find the difference at 5% (P ≤ 0.05) level. Simultaneously, the percentage survival of spawn in the different groups of each challenge treatment was recorded daily over 7 days.

#### Results

There were changes in ACP activity, total protein content, lysozyme activity, bactericidal activity and superoxide anion assay in different glucan treated/ untreated groups of *A testudineus* spawns, though the number of leucocytes were adjusted to a fixed level of 10^5^ cells ml^-1^.

**Figure 1:**
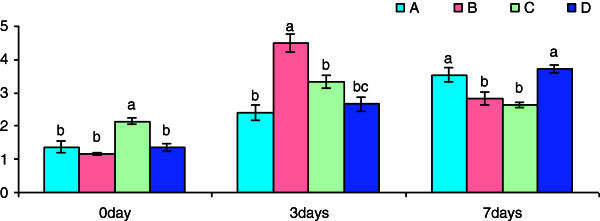
**Pre-challenge and post-challenge total protein (mgml**^**-1**^**) content of the cell suspension collected after different time of exposure with betaglucan.** Bars represent pooled mean values. **A**, 0 mgl^-1^ glucan: **B**, 5 mgl^-1^ glucan: **C**, 10 mgl^-1^ glucan: **D**, 15 mgl^-1^ glucan. Bars bearing common superscript are not significant at 5% level in comparison to each other (n = 6).

The total protein content of cell suspension lysate was significantly increased in Group C fish after 3 h β-glucan post-treatment (Figure 1). There was significant increase in protein content in Groups B and C after day 3 post-challenge with *S. paracitica.* However, the protein content was reduced significantly in Groups B and C after day 5 post-challenge. In case of group D fish the protein content was higher than the control group of fish.

**Figure 2:**
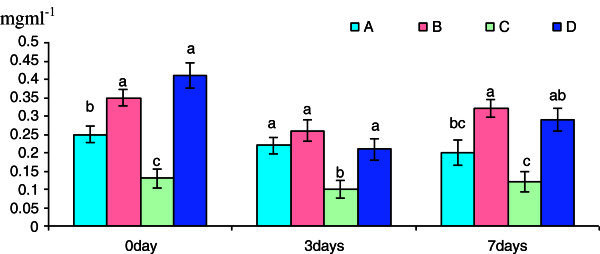
**Pre-challenge and post-challenge lysozyme (Ul**^**-1**^**) content of the cell suspension collected after different time of exposure with betaglucan.** Bars represent pooled mean values. **A**, 0 mgl^-1^ glucan: **B**, 5 mgl^-1^ glucan: **C**, 10 mgl^-1^ glucan: **D**, 15 mgl^-1^ glucan. Bars bearing common superscript are not significant at 5% level in comparison to each other (n = 6).

The lysozyme enzyme activity was significantly increased in groups B and D after 3h exposure to β-glucan over control. There was a significant increase in lysozyme activity in Group B and D over the control on day 5 post-challenge treatment with *S. parasitica* (Figure 2). However, the lysozyme enzyme activity was significantly decreased in Group C invariable of treatment with β-glucan and post- challenge with *S. parasitica.*

**Figure 3:**
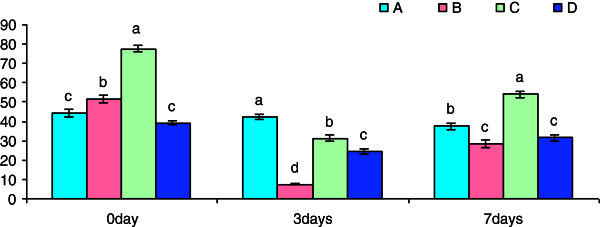
**Pre-challenge and post-challenge bactricidal (%) activity of the cell suspension collected after different time of exposure with betaglucan.** Bars represent pooled mean values. **A**, 0 mgl^-1^ glucan: **B**, 5 mgl^-1^ glucan: **C**, 10 mgl^-1^ glucan: **D**, 15 mgl^-1^ glucan. Bars bearing common superscript are not significant at 5% level in comparison to each other (n = 6).

A significant increase in bactericidal activity was observed in Groups B and C exposed for 3 h in β-glucan suspension as compared to control (Figure 3). However, the bactericidal activity was significantly reduced in all the groups on day 3 and day 5 of post-challenge treatment with *S. parasitica* over the control group expect for Group C on day 5 post-challenge treatment which showed a significant increase in bactericidal activity.

**Figure 4:**
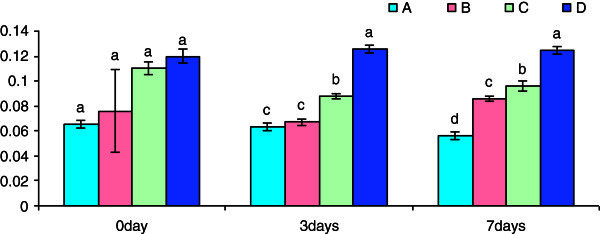
**Pre-challenge and post-challenge NBT (O.D.) activity of the cell suspension collected after different time of exposure with beta-glucan.** Bars represent pooled mean values. **A**, 0 mgl^-1^ glucan: **B**, 5 mgl^-1^ glucan: **C**, 10 mgl^-1^ glucan: **D**, 15 mgl^-1^ glucan. Bars bearing common superscript are not significant at 5% level in comparison to each other (n = 6).

The leucocyte cell suspension of spawns of *Anabas* showed a marginal increase in superoxide anion activity in groups exposed to 3 h β-glucan over the control group. There was a significant increase in superoxide anion activity in Groups C and D on day 3 post-challenge treatment with *S. parasitica.* Similarly, significant increase was observed in all the Groups (B,C, D) on day 5 post-challenge treatment with *S. parasitica* (Figure 4)*.*

**Figure 5:**
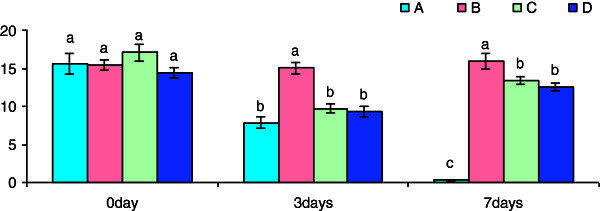
**Pre-challenge and post-challenge acid phosphatase (Ul**^**-1**^**) activity of the cell suspension collected after different time of exposure with beta-glucan.** Bars represent pooled mean values. **A**, 0 mgl^-1^ glucan: **B**, 5 mgl^-1^ glucan: **C**, 10 mgl^-1^ glucan: **D**, 15 mgl^-1^ glucan. Bars bearing common superscript are not significant at 5% level in comparison to each other (n = 6).

The AcP activity recorded a marginal increase in Group C (9.83%) over the control group after 3 h β-glucan exposure in pre-challenge treatment. There was significant increase in the AcP activity in Group B on day 3 post-challenge treatment with *S. parasitica.* Groups C and D recorded an increase of 23.75% and 19.28% respectively over the control group A on day 3 post-challenge treatment (Figure 5). The AcP activity was significantly increased in all groups (B,C,D) on day 5 post-challenge treatment over the control group A.

**Figure 6:**
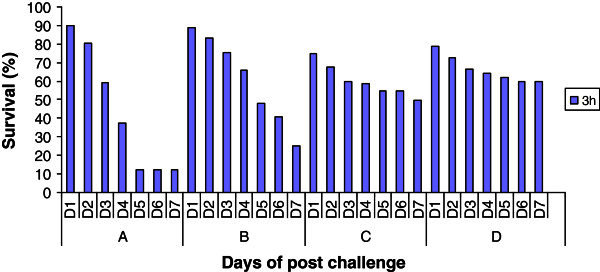
**Percent survival of spawn after immersion challenge with*Saprolegnia parasitica*(n = 32). A**, 0 mgl^-1^ glucan: **B**, 5 mgl^-1^ glucan: **C**, 10 mgl^-1^ glucan: **D**, 15 mgl^-1^ glucan.

The survivability percentage of post challenge treatment group is indicated in Figure 6. It was noticed that survival percentage was maximum in Group D fish exposed to 3 h post immersion with β–glucan suspension after day 7 (Figure 6). Survivability was only 12.5 percent in control groups.

## Discussion

The present work demonstrates the effect of multiple immersion dosages of β-glucan suspension on immune response and disease resistance in *A. testudineus* spawns against a fungal pathogen *Saprolegnia parasitica*.

As a first line of defense, mucous, epithelium from skin, gills and intestine and various peptides, lysozomal enzymes, complement factors and other lytic factors present in the serum prevent adherence and colonization of microorganisms (Alexander & Ingram 1992), resulting in the prevention of infection and diseases. In addition, macrophages which are innate immune cells present at hatching stage (Baulny et al. 1996) and innate immune factors constitute a second barrier against invading pathogens. The enhancements of lysozyme activity complement activity and bactericidal activity due to administration of β-glucan for a longer duration have been reported (Trinder 1969 Doumas et al. 1971 Bradford 1976 Dalmo et al. 1996). The effect of β-glucan on immunity and survival of *Anabas testudineus* against *A. hydrophila* was studied by Das et al. (2009). Based on previous observations, for exposure durations of 2 h and 3 h, it was found that the exposure of *A. testudineus* spawn to β-glucan at 10mgl^-1^ for 3 h followed by challenge with *A. hydrophila* provided better immunity and protection (Das et al. 2009). The present study was undertaken to document, the effect of β-glucan suspension on immune response and disease resistance of early stages of fish against fungal pathogen *S. parasitica*.

It has been shown that injection of laminarina β-1, 3 glucan typical of those present in fungal cell walls, induces immune responses in both adult and larval locusts (Goldsworthy et al. 2002; Mullen and Goldsworthy 2003). According to them, the activation of immune response varies in emerging adult as that of mature adults.

The total protein content of the cell suspension was found to change upon exposure to β glucan suspension of various concentrations over 3 h exposures as reported by Das et al. (2009). Following challenge with *S. parasitica*, the protein concentration was increased in all the groups as compared to control on day 3 and decreased significantly in groups B and C on day 7.

There was increase in lysozyme activity in all the treatment groups as compared to the control as reported in our previous work (Das et al. 2009). The post- challenge groups of fishes showed increased lysozomal enzymes but the increase was marginal as compared to challenge with *A. hydrophila*(Das et al. 2009). This indicates that lysozomal enzymes produced by the leucocytic cell suspension may be spared to counter act the fungal challenge.

Bactericidal activity of cell suspension was also increased upon exposure to 3 h β-glucan suspension at all doses but decreased followed by post challenge fish on day 3 and day 7 except experimental group C on day 7. Increased bactericidal activity was reported by Das et al. (2009) following β-glucan immunostimulation. Interestingly, *A. hydrophila* post-challenge increased the bactericidal activity (Das et al. 2009) and *S. parasitica* post-challenge decreased the bactericidal activity. Such decreased bactericidal properties of fish cell suspension involved in some innate and/or adaptive immune response are depressed in the cell suspension at the early stages which are utilized to fight against fungal infection.

Interestingly there is a rise in total NBT positive cells after exposure of β-glucan suspension for 3 h followed by fungal infection as reported with bacterial challenge (Das et al. 2009). Misra *et al.*(2005) reported a peak O_2_^-^ production during the NBT assay after feeding β–glucan at a dose of 500 mg kg^-1^ diet for 42 days. The role of β-glucan in our study was visible from the mortality pattern in the spawn for 7 days post challenge with *S. parasitica* which could be attributed to the increased activities of different peptides (Couso et al. 2003; Misra et al. 2005). Post challenged group showed increased phosphatase activities indicates higher breakdown of energy reserves, which was utilized by the growth and survival of fish (Das et al. 2009). Though, the acidphosphatase activity was decreased in prechallenged glucan stimulation at 5 and 15 mg ml^-1^ which might be corroborated with the findings of Sahu et al. (2008) and Das et al. (2009).

Exposure of β-glucan at different dosages in our study showed reduced mortality on the fish on the 7th day post challenge through bath immersion with the fungal *pathogen S. parasitica*. The protective effect of β-glucan against the fungus noticed in the challenge study irrespective of dosages. Further, it can be inferred from the bath challenge study that the increased protection against the fungus could be due to the enhanced immunity as evidenced by the increase in different immune parameters of the leucocytes cell suspension.

It is thus evident from the present experiment that β-glucan also protect the fish from fungal infection of *S. parasitica* at the 10 and 15 mgl^-1^ concentrations exposure for 3 h as noticed from the survival rate. But in earlier work, we have reported that β-glucan at a dose of 10 mgl^-1^ for 3 h is sufficient enough for giving protection against *Aeromonas hydrophila* infection. Though 15 mgl^-1^ gives little higher survival but 10 mgl^-1^ is equally good enough for protection (60% and 50% respectively). Especially spawn of 8 days old receives protection to both the bacteria and fungi and increased nonspecific immunity as evidenced from the leucocytes suspension parameters. This will have a great impact in the nursery phase rearing of this species where spawns could be exposed to glucan suspension for preventing loss due to fungal and bacterial infections and will provide healthy spawn for grow out culture practices to the farmers.
